# Characterization of the complete mitochondrial genome and phylogenetic analysis of the common dolphin *Delphinus delphis* (Cetacea: Delphinidae)

**DOI:** 10.1080/23802359.2018.1473720

**Published:** 2018-05-23

**Authors:** Kyunglee Lee, JunMo Lee, Yuna Cho, Hawsun Sohn, Young-Min Choi, Se Ra Lim, Hye Kwon Kim, Sun-Woo Yoon, Dae Gwin Jeong, Ji Hyung Kim

**Affiliations:** a Cetacean Research Institute (CRI), National Institute of Fisheries Science (NIFS), Ulsan, Republic of Korea;; b Department of Biological Sciences, College of Science, Sungkyunkwan University, Suwon, Republic of Korea;; c Infectious Disease Research Center, Korea Research Institute of Bioscience and Biotechnology, Daejeon, Republic of Korea;; d Bio-Analytical Science Division, University of Science and Technology (UST), Daejeon, Republic of Korea

**Keywords:** *Delphinus delphis*, mitogenome, phylogeny, Delphininae

## Abstract

We report the complete mitogenome of the common dolphin, *Delphinus delphis*. Overall structure of the 16,387 bp mitogenome was very similar to those of other delphinid species, including the ancient *D. delphis* individuals. Multigene phylogeny revealed that *D. delphis* was most closely related to Stenella coeruleoalba, and clustered well with other species within the subfamily Delphininae.

Although the genus Delphinus (Linnaeus, 1758) known to contain two species, the long-beaked form (*D. capensis*) is currently synonymized with *D. delphis* (Perrin 2018). This species is widely distributed in the Atlantic and Pacific Oceans with two distinct subpopulations in the Mediterranean and Black Sea (Perrin 2002; Hammond et al. 2008). At present, *D. delphis* is listed in Appendix II of the Convention on International Trade in Endangered Species (CITES 2014) and has been listed as ‘Least Concern’ by the International Union for Conservation of Nature and Natural Resources Red List of Threatened Species (Hammond et al. 2008). A recent study revealed several mitogenomes of ancient *D. delphis* individuals excavated in the Black sea (Biard et al. 2017); however, the mitogenomes from existing individuals have not yet been reported. We report the complete mitogenome of an existing *D. delphis* individual.

Muscle tissue sample was collected from the frozen carcass of an adult male *D. delphis* (voucher no. CRI007520) deposited in the Cetacean Research Institute, National Institute of Fisheries Science that was found lifeless among the bycatch of gillnet fisheries in Mar 2017 along the South Sea (South Korea, 33°13′25″N 126°25′42″E). The *D. delphis* mitogenome was obtained by designating 15 primer pairs from the mitogenome of *D. capensis* (NC_012061.1) (Lee et al. 2018), and the sequences obtained using PCR amplifications were assembled and annotated as previously described (Kim et al. 2017).

The *D. delphis* mitogenome (MH000365) was 16,387 bp in length (61.3% A + T content), and included a typical set of 37 genes (two rRNAs, 22 tRNAs, and 13 PCGs), and overall gene arrangement and content of the mitogenome were almost identical to those from other related Delphinidae species. The mitogenomic relatedness between *D. delphis* and other Delphinidae species was respectively determined using the ANI calculator (http://www.ezbiocloud.net/tools/ani). Although the mitogenomes from two ancient *D. delphis* individuals (Biard et al. 2017) were the most similar to CRI007520 with 99.6% each, the highest species-specific OrthoANI value was obtained for Stenellacoeruleoalba (NC_012053, 98.3%), whereas the lowest was for killer whale (*Orcinus orca*; NC_023889, 93.0%).

The taxonomical position of *D. delphis* was determined using multigene phylogenetic analysis (Kim et al. 2017). The resultant phylogeny suggested that *D. delphis* was most related to *S. coeruleoalba* and clustered well with other dolphin species within the subfamily Delphininae (Rosel et al. 1994; LeDuc et al. 1999; Amaral et al. 2007; McGowen 2011; Amaral et al. 2012) (Figure 1). The first decoded complete mitogenome of *D. delphis* and its associated genomic information might provide important insights for its conservation and further evolutionary studies within the subfamily Delphininae.

**Figure 1.**
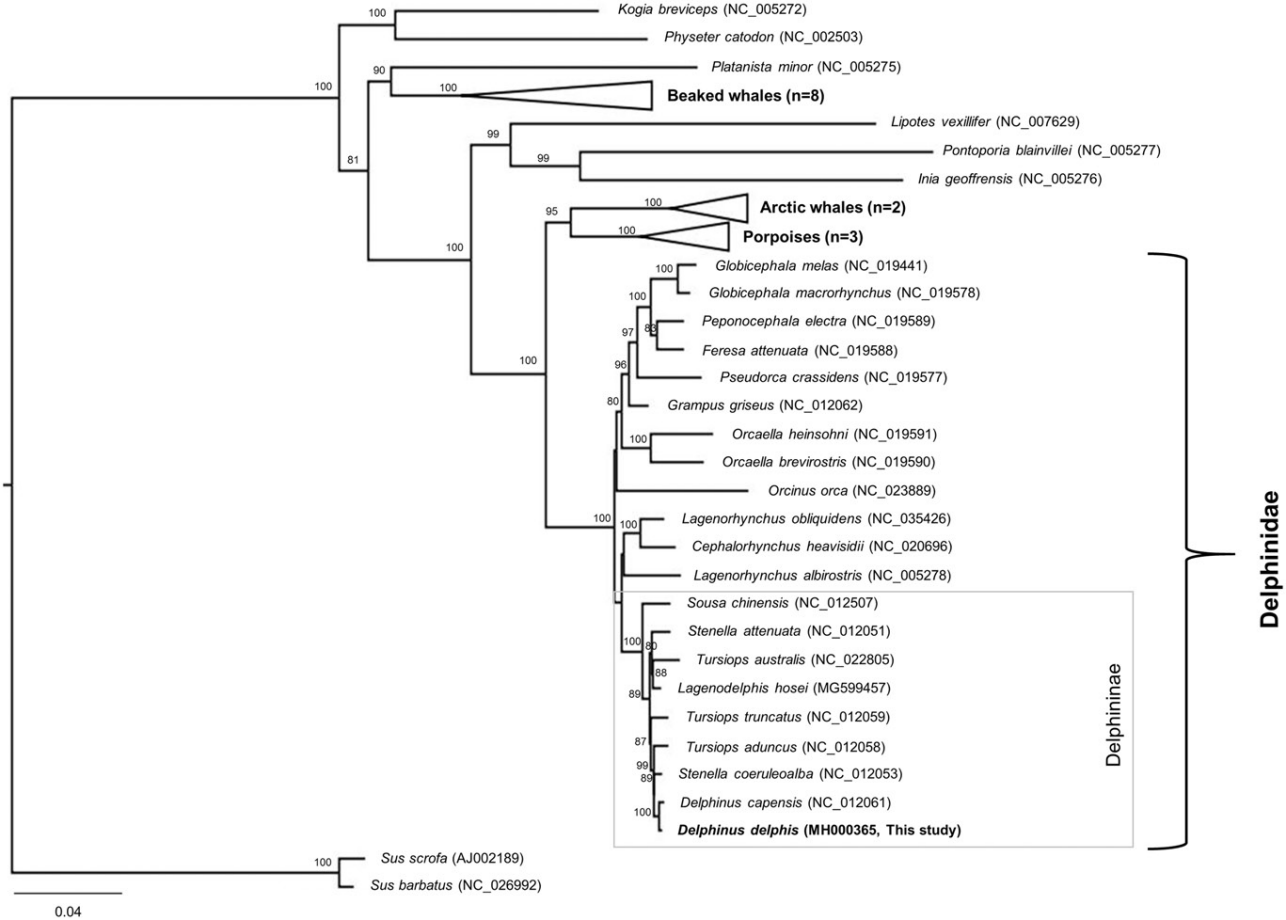
Maximum-likelihood phylogeny based on the 13 concatenated PCGs from the available Odontoceti mitogenomes. The grey square box denotes the species currently placed in the subfamily Delphininae. Numbers at the branches indicate bootstrapping values obtained with 1000 replicates, and only bootstrap values >70% are indicated. The scale bar represents 0.04 nucleotide substitutions per site. *D. capensis* is currently synonymized with *D. delphis*.
